# Molecular subtyping for clinically defined breast cancer subgroups

**DOI:** 10.1186/s13058-015-0520-4

**Published:** 2015-02-26

**Authors:** Xi Zhao, Einar Andreas Rødland, Robert Tibshirani, Sylvia Plevritis

**Affiliations:** Center for Cancer Systems Biology, Stanford University, James H. Clark Center 318 Campus Drive, Stanford, CA 94035-5442 USA; Department of Radiology, School of Medicine, Stanford University, James H. Clark Center, Room S255, 318 Campus Drive, Stanford, CA 94305-5442 USA; Department of Tumor Biology, Institute for Cancer Research, The Norwegian Radium Hospital, Oslo University Hospital, Oslo, 0424 Norway; Departments of Health, Research & Policy, and Statistics, Stanford University, Redwood Building, Room T101C, 150 Governor’s Lane, Stanford, CA 94305 USA

## Abstract

**Introduction:**

Breast cancer is commonly classified into intrinsic molecular subtypes. Standard gene centering is routinely done prior to molecular subtyping, but it can produce inaccurate classifications when the distribution of clinicopathological characteristics in the study cohort differs from that of the training cohort used to derive the classifier.

**Methods:**

We propose a subgroup-specific gene-centering method to perform molecular subtyping on a study cohort that has a skewed distribution of clinicopathological characteristics relative to the training cohort. On such a study cohort, we center each gene on a specified percentile, where the percentile is determined from a subgroup of the training cohort with clinicopathological characteristics similar to the study cohort. We demonstrate our method using the PAM50 classifier and its associated University of North Carolina (UNC) training cohort. We considered study cohorts with skewed clinicopathological characteristics, including subgroups composed of a single prototypic subtype of the UNC-PAM50 training cohort (n = 139), an external estrogen receptor (ER)-positive cohort (n = 48) and an external triple-negative cohort (n = 77).

**Results:**

Subgroup-specific gene centering improved prediction performance with the accuracies between 77% and 100%, compared to accuracies between 17% and 33% from standard gene centering, when applied to the prototypic tumor subsets of the PAM50 training cohort. It reduced classification error rates on the ER-positive (11% versus 28%; *P* = 0.0389), the ER-negative (5% versus 41%; *P* < 0.0001) and the triple-negative (11% versus 56%; *P* = 0.1336) subgroups of the PAM50 training cohort. In addition, it produced higher accuracy for subtyping study cohorts composed of varying proportions of ER-positive versus ER-negative cases. Finally, it increased the percentage of assigned luminal subtypes on the external ER-positive cohort and basal-like subtype on the external triple-negative cohort.

**Conclusions:**

Gene centering is often necessary to accurately apply a molecular subtype classifier. Compared with standard gene centering, our proposed subgroup-specific gene centering produced more accurate molecular subtype assignments in a study cohort with skewed clinicopathological characteristics relative to the training cohort.

**Electronic supplementary material:**

The online version of this article (doi:10.1186/s13058-015-0520-4) contains supplementary material, which is available to authorized users.

## Introduction

Breast cancer is intrinsically heterogeneous. On the basis of gene expression, breast tumors are often classified as one of five intrinsic subtypes, luminal A (LumA), luminal B (LumB), human epidermal growth factor receptor 2 (HER2)-enriched, basal-like and normal-like [1-3]. The original intrinsic subtype classification was obtained through unsupervised clustering of breast tumors based on the expression of the “intrinsic” gene set selected for small intratumor variation, before and after neoadjuvant chemotherapy, and large intertumor variation [1]. Variants using different gene sets have emerged [2-6]. Generally, these molecular subtypes are associated with distinct biological features and clinical outcomes. They contribute to insights into cancer initiation and progression and could guide clinical decisions [2,3,6-8]. Hence, high accuracy of molecular subtyping is critical.

The molecular subtype of an individual breast tumor drawn from a study cohort is typically assigned to its closest matching subtype expression profile. To increase specificity, each molecular subtype is defined by a centroid based on gene expression values in the training cohort. A tumor is assigned to the molecular subtype that has the highest correlation between the subtype’s centroid and the study tumor’s corresponding gene expression pattern [2-4,6]. Gene expression levels vary greatly because of technical biases, so it is necessary to center the gene expression values in the study cohort prior to subtyping [9]. Standard gene centering is typically done by subtracting the median or mean expression per probe or gene across the study cohort. However, standard gene centering introduces errors in molecular subtype classifications when the clinicopathological distributions of the study cohort do not match those of the training cohort used to derive the molecular subtype classifier.

Training cohorts used to develop the subtype expression signature are often intended to represent the general patient population [1-3,6]. For PAM50 [6] classifiers, the original training cohort, referred to as the University of North Carolina (UNC) dataset, is regarded as capturing the major breast cancer types in the general patient population in their relative proportions. When using the intrinsic subtype classifier, the clinicopathological distribution of breast cancer of the study cohort should be similar to that of the training cohort [9]. For study and training cohorts with similar clinicopathological distributions, systematic differences in gene expression are assumed to be due to technical biases; after standard gene centering to remove these technical biases, the cohorts are considered comparable. In the case of the PAM50 signature, the UNC dataset was centered probe-wise prior to establishment of the centroids for the subtypes.

**Figure 1:**
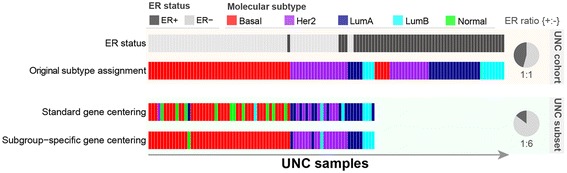
**Effect of estrogen receptor distribution on molecular subtype assignments.** The University of North Carolina (UNC) cohort is the PAM50 training cohort. Only samples with available prototypic tumor subtypes and available estrogen receptor (ER) status are shown (n = 118). In each horizontal strip, the vertical bands represent individual patients and are arranged in the same sequence for each horizontal band. First, we considered the UNC cohort, where there was a balanced ER-positive to ER-negative distribution—46% ER-positive (54/118) and 54% ER-negative (64/118)—represented by the shaded pie chart labeled “UNC cohort.” In the first strip at the top, labeled “ER status”, the ER status on the UNC cohort is depicted as dark vs. light gray, representing ER-positive vs. ER-negative cases, respectively. In the second strip, labeled “Original subtype assignment,” the original subtype assignments on the UNC cohort are shown. Next, we considered a subset of the UNC cohort (n = 75), which we created by sampling ER-positive and ER-negative cases disproportionally, with 15% ER-positive (11/75) and 85% ER-negative (64/75), as represented by the pie chart labeled “UNC subset.” In the third strip, labeled “Standard gene centering,” assigned subtypes by standard gene centering on the subset of the UNC subset, where ER is disproportionally distributed, are shown. The misclassification rate is 33.3% (25/75) compared with the first 75 bands in the second strip. In the bottom strip, labeled “Subgroup-specific gene centering,” assigned subtypes by the proposed subgroup-specific gene centering on the subset of the UNC cohort, where ER is disproportionally distributed, are shown. The misclassification rate is 5.3% (4/75). Here the classification is similar to the actual classification, shown in the first 75 cases of the second strip, labeled “Original subtype assignment.” Her2, Human epidermal growth factor receptor 2; LumA, Luminal A; LumB, Luminal B.

Although standard gene centering is commonly applied prior to molecular subtyping, it produces inaccurate molecular subtype classifications when the study and training cohorts differ in their clinicopathological composition. In particular, if the training cohort is intended to capture the heterogeneity of the general patient population, standard gene centering will not produce molecular subtype classifications on a study cohort with more narrowly defined clinicopathological characteristics relative to the general population, such as a study cohort of only ER-positive cases. More generally, if one were subtyping using standard gene centering and evaluating the assignments by ER status, the assignment to each ER-positive case would depend on how many ER-negative cases are in the study cohort. Similarly, the assignment to the ER-negative cases would depend on how many ER-positive are in the study cohort (Figure 1). The most accurate assignment would occur only when the proportion of ER-positive to ER-negative cases was similar to that of the training cohort; otherwise, errors would occur, as we demonstrate in this article.

A few approaches have been proposed to remedy the problem of applying standard gene centering when the study and training cohorts differ in terms of clinicopathological distribution. One can augment the study cohort with additional samples to form a new study cohort that better matches the heterogeneity of the training cohort. However, this approach assumes the availability of additional samples profiled on the same platform. Alternatively, one might consider subsetting or resampling the study cohort to match the training cohort in terms of clinicopathological characteristics [10,11]. However, subsetting makes inefficient use of the study data by removing samples, whereas resampling introduces randomness through the sampling scheme. Moreover, both of these approaches assume that the study cohort contains some fraction of all the subgroups in the training cohort, which would not be the case if the study cohort were only ER-positive patients, for example. Model-based methods (for example, distance-weighted discrimination [12]) have been proposed to remove technical bias while keeping biologically relevant signals; for these approaches, one should be cautious about the relevance of the underlying modeling assumptions.

To perform subtype classification on a study cohort whose clinicopathological composition differs from the training cohort, we propose *subgroup-specific gene centering* that corrects for these differences. Our method is a probe-wise, platform-independent, model-free strategy for subtype classification that does not rely on altering the study cohort by augmenting, removing or resampling it.

## Methods

For illustration purposes, we compare our subgroup-specific gene centering with standard gene centering using the PAM50 classifier. We refer to the *baseline expression* of a gene as the value against which that gene is centered. For standard gene centering, all probes are median-centered prior to classification by PAM50. Another subtyping method may define the baseline value differently (for example, it may be the mean); regardless, our approach, as presented below, would still apply.

### Formulation of subgroup-specific gene centering

**Figure 2:**
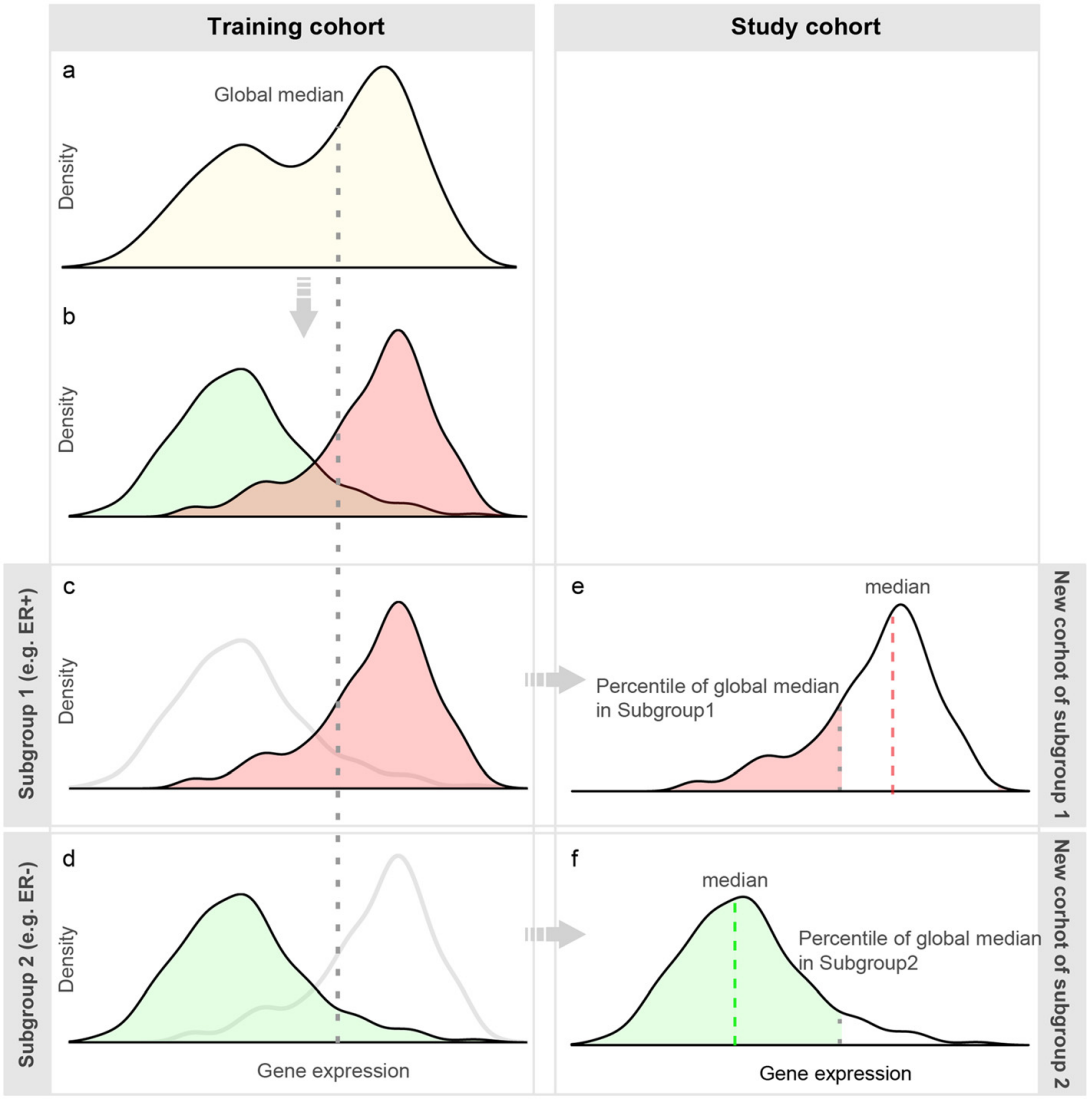
**Overview of subgroup-specific gene-centering algorithm. (a)** Distribution of gene expression for a representative gene from the entire University of North Carolina (UNC) training cohort, with the global mean represented by the gray vertical dotted line. **(b)** The gene expression baseline is approximated by the global mean (gray dotted line) shown on the global distribution, represented as a mixture of estrogen receptor (ER)-positive cases (shown in pink) and ER-negative cases (shown in green). **(c)** and **(d)** The global median is located on different percentiles for the ER-positive and ER-negative cases, and each differs with respect to each subgroup mean. **(e)** The distribution of gene expression for the same gene in a study cohort composed of only ER-positive cases. The baseline value for subgroup-specific gene centering is estimated at the corresponding percentile of the ER-positive subgroup in the study cohort and compared with the median value, represented by the red vertical dotted line. The difference between these values is the error introduced by standard gene centering. **(f)** Similar to **(e)**, but for the ER-negative subgroup.

We propose a subgroup-specific centering method, illustrated in Figure 2, to replace standard gene centering when subtyping a study cohort that differs from the training cohort in terms of its clinicopathological distribution. The steps illustrated in Figure 2 are performed on each gene of the gene expression signature, which is the PAM50 signature in this case.

In the training cohort, we compute the expression baseline, μ_*gene*_, of each gene. In PAM50, this is the median gene expression across the cohort. In standard gene centering, μ_*gene*_ is subtracted from the expression value of that gene.

In our approach, prior to classification, we sample a subgroup of the training cohort with a clinicopathological distribution similar to that of the study cohort. For this subgroup, we find the expression value at the percentile *Q*_*gene*_ that corresponds to the percentile of the baseline expression μ_*gene*_ of the entire training cohort. In other words, we estimate where the baseline expression μ_*gene*_ of the entire training cohort lies within the study cohort. We then assign the baseline expression of each gene at the *Q*_*gene*_ percentile and center the gene expression by subtracting the value at this percentile. Additional file 1: Table S6 lists baseline values precomputed for common breast cancer subgroups, specifically ER-positive, ER-negative and triple-negative cases.

For example, consider the gene *ESR1*, whose median expression value is 1.68 in the UNC training cohort and lies at 20th percentile for the ER-positive subgroup of the training cohort. If one were molecularly subtyping the ER-positive-only study cohort published by Borgan *et al*. [13], the expression value of *ESR1* would be adjusted by subtracting its median value of 3.34 using standard gene centering. Using our approach, the expression of *ESR1* is centered on the value corresponding to the 20th percentile for the study cohort, which is 1.3 (see Additional file 2: Figure S1). Below we show that our approach produces fewer errors for molecular subtype classification.

If the study cohort were a mixture of several subgroups (for example, a mixture of 15% ER-positive and 85% ER-negative samples), the gene-specific percentiles could be computed based on a sampling of the training cohort with a similar mixture.

Subgroup-specific gene centering was implemented in R (version 3.0.0) [14]. Data and code were deposited in the Stanford Center for Cancer Systems Biology Data Integration Core database [15]. The supplementary files provide statistical and implementation details.

### Evaluation of subgroup-specific gene centering and associated datasets

We demonstrate the improved performance of subgroup-specific versus standard gene centering on two types of study cohorts. First, we considered study cohorts derived from the UNC dataset, where the “true” (or prototypic) subtypes for the tumors were known but blinded from our classifier. Second, we considered two external study cohorts: namely, cohorts independent of the UNC dataset used to develop the PAM50 classifier. One external cohort was composed of only ER-positive tumors and the other of only triple-negative breast cancer (TNBC). All the data analyzed in this study were previously published. Ethical approval was not required because no human breast tissue was acquired for this study.

#### Evaluation of subgroup-specific strategy on study cohorts derived from the PAM50 training cohort

For the purposes of quantifying the accuracy of subgroup-specific versus standard gene centering, we derived study cohorts from subgroups of the UNC dataset [GEO:GSE10886] (see also Additional file 3: Supplementary methods). We considered the five UNC prototypic tumor subgroups: the prototypic LumA set, the prototypic LumB set, the prototypic HER2-enriched set, the prototypic basal-like set and the prototypic normal-like set. These were used to define the average expression profiles (centroids) for each of the molecular subtypes. In addition, we considered three clinically defined tumor subgroups of the UNC dataset, namely: ER-positive, ER-negative and triple-negative subsets. Here the UNC dataset was regarded as a representative cohort of breast cancer, and prototypic labels of the UNC samples were considered the true molecular subtypes when computing the subtyping accuracy. To assess classification accuracy, the prototypic subtypes for the tumors were known but blinded from the classifier. The classification accuracy was calculated as the percentage of samples with predicted subtype matching their prototypic labels, and the misclassification percentage was the error rate.

#### Evaluation of subgroup-specific strategy on external study cohorts

##### Triple-negative cohort

The TNBC dataset (n = 77) is a subset of The Cancer Genome Atlas (TCGA) breast data [11] with negative immunohistochemical expression for ER, progesterone receptor and HER2. This TCGA level 3 dataset, its clinical annotation and the published molecular subtype calls were obtained through the TCGA website [16].

##### Estrogen receptor-positive cohort

The Trondheim set (n = 48) is a published dataset of ER-positive breast tumors from Trondheim, Norway [13]. The article by Borgan *et al*. [13] includes information for data preprocessing and normalization. The principal investigators of this study provided the data (see Acknowledgements) and stated that ethical approval was granted by the regional committee for medical and health research ethics (REC Central, Norway). The approval numbers are 4.2006.216 (before 2010) and 2010/331 (after 2010).

On these external study cohorts, associations between molecular subtypes with known clinicopathological features were used to assess the subtype assignments. Enrichment of luminal and the basal-like subtypes were assumed for the external ER-positive cohort (Trondheim set) and the TNBC dataset, respectively. In addition, for the external TNBC dataset, subgroup-specific and standard centering were benchmarked against the published PAM50 subtype classifications, which were generated using the full TCGA dataset.

## Results

### Evaluation of subgroup-specific strategy on study cohorts derived from the UNC dataset

#### Prototypic subgroups from the UNC dataset

**Figure 3:**
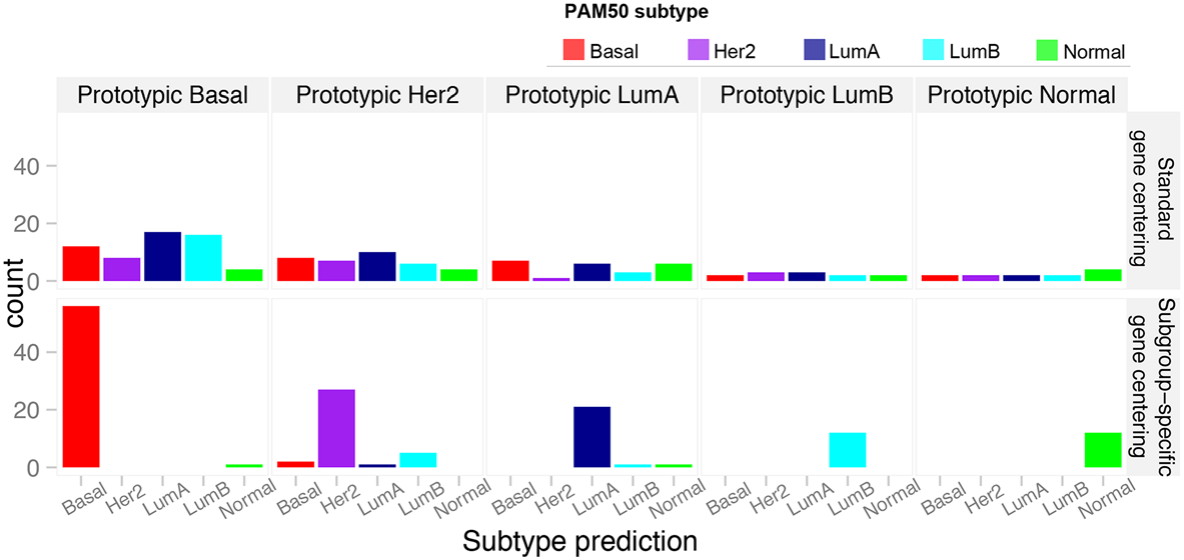
**Comparison of standard with subgroup-specific gene centering for predicting the individual molecular subtypes on the prototypic datasets.** Bar plot represents the counts of the predicted subtype classes in individual prototypic tumor dataset. Her2, Human epidermal growth factor 2; LumA, Luminal A; LumB, Luminal B.

We evaluated subgroup-specific versus standard gene centering for predicting the intrinsic subtypes on subgroups composed of single intrinsic subtypes from the UNC dataset (Table 1 and Figure 3). We assessed a molecular classification as correct if it matched the prototypic subtypes. With standard gene centering, the prediction accuracy range was 17% to 33% across the five intrinsic subtypes. Subgroup-specific gene centering produced accuracies in the range from 77.1% to 100%, with 98.2% (56/57) for basal-like, 77.1% (27/35) for HER2-enriched, 91.3% (21/23) for LumA, 100% for LumB (12/12) and 100% (12/12) for normal-like prototypic tumors.

**Table 1:** **Comparison of gene centering with a subgroup-specific strategy on the UNC prototypic tumor set** ^**a**^

			**Prediction (%)**				
**Dataset**	**Subgroup**	**Gene-centering method**	**Basal-like**	**HER2-enriched**	**LumA**	**LumB**	**Normal-like**
Prototypic basal	Basal-like	Standard	12 (21.1)	8 (14)	17 (29.8)	16 (28.1)	4 (7)
(n = 57)		Subgroup-specific	56 (98.2)	0 (0)	0 (0)	0 (0)	1 (1.8)
Prototypic HER2	Her2-enriched	Standard	8 (22.9)	7 (20)	10 (28.6)	6 (17.1)	4 (11.4)
(n = 35)		Subgroup-specific	2 (5.7)	27 (77.1)	1 (2.9)	5 (14.3)	0 (0)
Prototypic LumA	Luminal A	Standard	7 (30.4)	1 (4.3)	6 (26.1)	3 (13)	6 (26.1)
(n = 23)		Subgroup-specific	0 (0)	0 (0)	21 (91.3)	1 (4.3)	1 (4.3)
Prototypic LumB	Luminal B	Standard	2 (16.7)	3 (25)	3 (25)	2 (16.7)	2 (16.7)
(n = 12)		Subgroup-specific	0 (0)	0 (0)	0 (0)	12 (100)	0 (0)
Prototypic normal	Normal-like	Standard	2 (16.7)	2 (16.7)	2 (16.7)	2 (16.7)	4 (33.3)
(n = 12)		Subgroup-specific	0 (0)	0 (0)	0 (0)	0 (0)	12 (100)

^a^HER2, Human epidermal growth factor receptor 2; LumA, Luminal A; LumB, Luminal B; UNC, University of North Carolina.

#### Clinically defined subgroups from the UNC dataset

Next, we evaluated the subtyping accuracy on the ER-positive, ER-negative and triple-negative subgroups of the UNC dataset. On the ER-positive subgroup (Additional file 1: Table S2), the error rate was 11% (6/54) versus 27.8% (15/54) error by subgroup-specific versus standard gene centering, respectively (*P* = 0.0389 by McNemar test). Our method mostly misclassified prototypical HER2-enriched samples. Four prototypic HER2-enriched samples were classified as LumB, and one was classified as basal-like. One prototypic LumA tumor was misclassified as LumB. On the ER-negative subgroup (Additional file 1: Table S3), our method versus the standard method produced an error rate of 5% (3/64) versus 41% (26/64), respectively (*P* < 0.0001 by McNemar test). With our method, only two prototypic HER2-enriched tumors were assigned to LumB and LumA, respectively, and one prototypic basal-like tumor was misclassified as normal-like. On the UNC triple-negative subgroup (Additional file 1: Table S4), our method produced an 11% (1/9) error rate, with one prototypic basal-like tumor incorrectly classified into normal-like, compared with a 56% (5/9) error rate by standard gene centering (*P* = 0.1336 by McNemar test; note that the small sample size, n = 9, is likely insufficient to detect a statistically significant difference). In supplementary work, we assessed the subtyping accuracy using our subgroup-specific strategy as a function of the sample size of the prototypic subgroup, shown for the basal subgroup in Additional file 4: Figure S3.

#### Mixture of ER-positive and ER-negative subgroups from the UNC dataset

**Figure 4:**
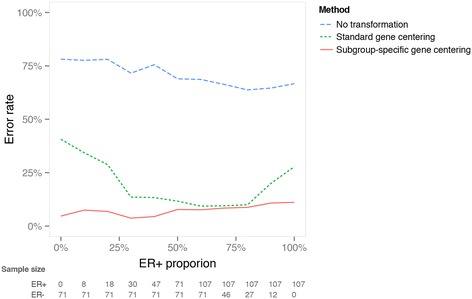
**Comparison of various data transformation strategies for predicting molecular subtypes on study cohorts with varying estrogen receptor proportions.** Datasets were constructed with percentages of estrogen receptor (ER)-positive cases ranging from of 0% to 100%. The ER-positive and ER-negative samples randomly drawn from the University of North Carolina set. Error rate is plotted against the composition with respect to ER for no, standard and subgroup-specific gene-centering strategies.

We assessed the subtype prediction performance on subgroups constructed with a varying percentage of ER-positive samples, gradually increasing from 0% to 100% in 10% increments (Figure 4). To construct these subgroups, samples were randomly drawn from the UNC dataset to achieve the specified ER distributions. Subgroup-specific gene centering was done after sampling a subgroup of the UNC set with a similar mixture. Its error rate was less than 11% across various ER proportions. The performance of standard gene centering was comparable to our method only when the proportion of ER-positive was 50% to 80%; it produced high error rates otherwise. Applying PAM50 classification on data without any gene centering produced the highest error rate (Figure 4), confirming the importance of some form of gene centering prior to subtyping.

### Application of subgroup-specific strategy on external study cohorts composed of common clinically relevant subgroups

In the Trondheim set (ER-positive cohort), individual genes were centered on the ER-positive-specific percentiles identified from the corresponding UNC subset (Additional file 1: Table S1 and Additional file 2: Figure S1). We expected the majority of the ER-positive cases would be luminal subtypes. With standard gene centering, only 43.8% tumors were assigned to luminal subtypes (18.8% (9/48) LumA and 25% (12/48) LumB). In contrast, our method produced 81.2% luminal subtypes, consisting of 47.9% (23/48) LumA and 33.3% LumB (16/48) (Table 2).

**Table 2:** **Comparison of gene centering with subgroup-specific strategy on the external study breast cohorts with skewed distribution** ^**a**^

			**Prediction (%)**				
**Dataset**	**Feature**	**Gene-centering method**	**Basal-like**	**HER2-enriched**	**LumA**	**LumB**	**Normal-like**
Trondheim	ER-positive	Standard	10 (20.8)	8 (16.7)	9 (18.8)	12 (25.0)	9 (18.8)
(n = 48)		Subgroup-specific	2 (4.2)	4 (8.3)	23 (47.9)	16 (33.3)	3 (6.2)
TNBC	Triple-negative	Standard	28 (36.4)	9 (11.7)	19 (24.7)	12 (15.6)	9 (11.7)
(n = 77)		Subgroup-specific	63 (81.8)	6 (7.8)	3 (3.9)	3 (3.9)	2 (2.6)

^a^ER, Estrogen receptor; LumA, Luminal A; LumB, Luminal B; TNBC, Triple-negative breast cancer.

On the TNBC-TCGA subgroup, we expected the majority of tumors would be basal-like. Subgroup-specific centering of the TNBC set on the triple-negative percentiles (Additional file 1: Table S1 and Additional file 5: Figure S2) resulted in 63 of the 77 TNBC tumors being classified as basal-like (82%), and the remainder were 6 HER2-enriched, 3 LumA, 3 LumB and 2 normal-like samples. With standard gene centering, only 36% were classified as the basal-like subtype (Table 2). The PAM50 subtypes published in the TCGA [11], generated using the full TCGA breast cohort, had 84% (65/77) of the cancers classified as basal-like (Additional file 1: Table S5). Compared with the published PAM50 subtypes, subgroup-specific versus standard gene centering produced error rates of 6.5% (5/77) versus 53.2% (41/77), respectively (*P* < 0.0001 by McNemar test).

## Discussion

Standard subtyping methods produced inaccurate classifications when the clinicopathological distributions of the study cohort did not match those of the training cohort. If one were to subtype a TNBC study cohort using the PAM50 classifier, the results would be an inaccurate representation of the PAM50 subtypes if standard gene centering was done, because the training cohort for the PAM50 classifier was intended to represent the general population, not the TNBC population. Although this problem is often overlooked, we are not the first to identify it. Others who have recognized the problem have proposed more complex solutions, including adding or removing samples from the study cohort to match the clinicopathological distribution of the training cohort. We propose a simpler solution because it does not involve altering the actual samples of the study cohort.

For accurate molecular subtyping, we present an alternative approach to gene centering that incorporates a routine data transformation step prior to subtyping. In standard gene centering, gene expression values are typically expressed relative to the median (or mean). We show that standard gene centering produces inaccurate molecular subtype classifications when the clinicopathological distribution of the study cohort differs from that of the training cohort. To address this issue, we propose a new method termed *subgroup-specific gene centering*, which adjusts gene expression values relative to the expression level at a specified percentile, where the percentile for each gene is determined from a subgroup of the training cohort with clinicopathological characteristics similar to those of the study cohort.

In comparison with standard gene centering, subgroup-specific gene centering more accurately reproduced the intrinsic subtypes when applied to the five prototypic tumor sets of the UNC cohort (Table 1 and Figure 3). Most notable were the accurate assignments to the luminal subtypes, where the differences in gene expression values defining LumA and LumB tumors are believed to be along a continuum [17]. This correct assignment indicates that these two types of luminal prototypic tumors in the UNC set are likely distinct in terms of their gene expression. Predictions on HER2-enriched prototypic tumors were relatively less accurate than the other prototypic sets using our method, and even less accurate using the standard method. The increased error in this subtype is likely due to its heterogeneous nature. On the ER-positive, ER-negative and triple-negative subgroups of the UNC cohort, our method produced lower classification error rates than standard gene centering. Moreover, subtype-specific centering was consistently more accurate than standard gene centering for study cohorts selected with varying portions of ER-positive and ER-negative cases.

Subgroup-specific gene centering performed well on the two external study cohorts consisting of ER-positive and TNBC tumors, respectively. It has been reported that ER-positive tumors tend be luminal, whereas at least 75% of TNBC cases are basal-like [17]. Subgroup-specific centering produced consistent findings, with 81% of the ER-positive tumors classified as luminal and 82% of the triple-negative tumors as basal-like. However, standard gene centering produced a more uniform distribution of the tumors between the five subtypes, with only 47% of the ER-positive tumors classified as luminal and 36% of the triple-negative tumors as basal-like. Although the information for triple-negative status is largely missing on the UNC dataset (only ten TNBCs available), our method outperforms the standard gene centering on this tumor subgroup as well.

The first step underlying our method is to find a subgroup of the training cohort that has clinicopathological characteristics similar to those of the study cohort. We demonstrate our method using ER status and TNBC status as the clinicopathological characteristics. Our method is quite flexible and can handle study cohorts composed of a mixture of several clinically defined subgroups. For illustrative purposes, we constructed study cohorts with different mixtures of ER-positive and ER-negative cases, because ER status is one of the major clinicopathological markers for breast cancer and well annotated in the UNC set. We then computed the gene-specific percentiles based on a subgroup of the training cohort with a similar mixture (for example, a UNC subset with a 15% to 85% mix of the ER-positive and ER-negative samples). Admittedly, this approach introduces some randomness because it involves sampling the corresponding mixture subgroups from the UNC set; however, the randomness can be averaged out by using a repeated sampling procedure. Alternatively, instead of subsampling, the samples may be given different weights based on their subgroup to match the composition of the study cohort.

Our method is not confined to a specific expression-profiling platform, and it does not require gathering additional samples to make the study cohort match the heterogeneity of the training cohort. In general, augmenting a dataset is subject to data availability and platform compatibility between study cohort and the additional samples. Platform differences often pose major obstacles for extracting biologically relevant signals while effectively removing technical biases.

Our subgroup-specific gene-centering strategy can be applied only when there are sufficient clinicopathological data to compare the study and training cohorts. If such data do not exist or if the data are highly correlated and do not capture sufficient heterogeneity to compare the study and training cohorts, our strategy cannot be applied. That said, there is no reassurance that standard gene centering is suitable when study and training cohorts cannot be compared.

Subgroup-specific gene centering is not suitable for study datasets with one patient sample, because it requires a cohort with sufficient sample size to ensure the subtyping accuracy. This limitation is largely technology-related, as expression measurements of microarrays are often analyzed on a relative rather than absolute scale, as well as in a platform-dependent manner. The data augmentation strategy by merging the single sample with a sizable cohort from a compatible platform is one solution under the current expression-based molecular subtyping. Alternatively, one can turn to techniques such as quantitative RT-PCR, which has technical advantages in terms of reproducibility and quantitative assessments [6,18,19].

Subgroup-specific gene centering has capability for immediate use for classifying molecular subtypes of breast cancer, which commonly include the intrinsic signature [1-3], PAM50 [6] and its extension to the claudin-low subtype [20]. In this article, we do not discuss the METABRIC study [10], because that study did not explicitly report a classifier (for example, centroids).

## Conclusions

Compared with standard gene centering, subgroup-specific gene centering enables more accurate molecular subtyping in a study cohort whose clinicopathological distribution does not match the training cohort. We demonstrate subgroup-specific gene centering the PAM50 breast cancer classifier, but emphasize that the subgroup-specific gene-centering approach is applicable to any classification based on gene-centered signatures. Moreover, it is not limited to breast cancer and can be applied to any tissue subtyping strategy reliant on a gene centering. It can also be applied to any characteristics for defining the subgroups, even mutational status, provided these are known in the training cohort.

## Additional files

Additional file 1: Table S1. Clinicopathological and molecular characteristics of UNC set (n = 232). **Table S2.** Subtype prediction by different strategies on UNC ER-positive subset (n = 107). **Table S3.** Subtype prediction by different strategies on UNC ER-negative subset (n = 71). **Table S4.** Subtype prediction by different strategies on UNC TN subset (n = 10). **Table S5.** Subtype prediction by different strategies on TCGA triple-negative subgroup (TNBC set; n = 77) compared with the published subtype calls on TCGA cohort. **Table S6.** PAM50 subgroup-specific gene centering baseline value for a few standard subgroups for breast tumors, computed using the UNC training set. Additional file 2: Figure S1. Subgroup-specific strategy for molecular subtype assignment in an ER-positive study cohort (Trondheim set) demonstrated for a single gene, *ESR1*. **(A)** Computing probe-wise subgroup percentiles. On the training cohort (UNC set), the global median (indicated by the gray dashed line) is mapped onto the distribution of ER-positive subgroup, and the corresponding percentile (20%) is the ER-positive-specific percentile used to adjust the expression of the gene *ESR1* in the study cohort. **(B)** Probe-wise transformation using subgroup-specific gene centering. On an external ER-positive study cohort (Trondheim set), instead of centering the gene value on the median of the study cohort, which is 3.34, as indicated by the red vertical dotted line), we center the gene *ESR1* around the 20th percentile of its distribution in the study cohort, which is 1.3. Additional file 3:
**Supplementary methods.** Molecular subtyping for clinically defined breast cancer subgroups. Additional file 4: Figure S3. Subtyping accuracy versus subgroup sample size of UNC basal prototypical subgroup. The horizontal axis denotes the sample size of data from downsampling UNC basal prototypical subgroup (from 57 to 1). The vertical axis shows the subtyping prediction accuracy using our subgroup-specific gene centering method. The accuracy is calculated based on the percentage of the predicted basal subtype on the corresponding dataset. Individual predictions are shown as circles, and a Loess smooth line (span 0.75) is fitted on the prediction points. Two hundred rounds of downsampling were performed. The color of the lines reflects the statistical confidence in the regression estimation. Additional file 5: Figure S2. Subgroup-specific strategy for molecular subtype assignment in a triple-negative breast cancer study cohort for the expression of gene *SLC39A6*. **(A)** Computing probe-wise subgroup percentiles. On the training cohort (UNC set), the global median of the UNC set (indicated by the white dashed line) is mapped onto the distribution of the TN subgroup, and the corresponding percentile (100%) is the TN-specific percentile for gene *SLC39A6*. **(B)** Probe-wise transformation using subgroup-specific percentile. On an external TNBC study cohort (TNBC set), instead of centering on the median of the study cohort (−1.46), we centered the distribution of gene *SLC39A6* on the 100% percentile of the TN distribution of the study cohort (3.98).

## Abbreviations

ER: Estrogen receptor
HER2: Human epidermal growth factor receptor 2
Intrinsic: Intrinsic signature
LumA: Luminal A
LumB: Luminal B
PAM50: PAM50 signature
TCGA: The Cancer Genome Atlas
TNBC: Triple-negative breast cancer
UNC: University of North Carolina
