# Supplementary material for: Molecular subtyping for clinically defined breast cancer subgroups
Source: Breast Cancer Res. 2015 Feb 26;17(1):29. doi: 10.1186/s13058-015-0520-4 (PMC4365540; doi:10.1186/s13058-015-0520-4)
Supplement: Additional file 1: Table S1. — Clinicopathological and molecular characteristics of UNC set (n = 232). Table S2. Subtype prediction by different strategies on UNC ER-positive subset (n = 107). Table S3. Subtype prediction by different strategies on UNC ER-negative subset (n = 71). Table S4. Subtype prediction by different strategies on UNC TN subset (n = 10). Table S5. Subtype prediction by different strategies on TCGA triple-negative subgroup (TNBC set; n = 77) compared with the published subtype calls on TCGA cohort. Table S6. PAM50 subgroup-specific gene centering baseline value for a few standard subgroups for breast tumors, computed using the UNC training set. [file 13058_2015_520_MOESM1_ESM.docx]

**Table S1. Clinicopathological and molecular characteristics of UNC set (n=232).**

| **Characteristics** |  | **n** | **%^a^** |
| --- | --- | --- | --- |
| All |  | 232 | 100 |
| ER (IHC) |  |  |  |
|  | Positive | 107 | 60.1 |
|  | Negative | 71 | 39.9 |
|  | Missing | 54 |  |
| HER2 (IHC) |  |  |  |
|  | Positive | 10 | 13.9 |
|  | Negative | 62 | 86.1 |
|  | Missing | 160 |  |
| PgR (IHC) |  |  |  |
|  | Positive | 41 | 60.3 |
|  | Negative | 27 | 39.7 |
|  | Missing | 164 |  |
| Triple negative (IHC) |  |  |  |
|  | Yes | 10 | 8.2 |
|  | No | 112 | 91.8 |
|  | Missing | 110 |  |
| Prototypic-subtype tumor |  |  |  |
|  | Prototypic Basal-like | 57 | 41 |
|  | Prototypic HER2-enriched | 35 | 25.2 |
|  | Prototypic LumA | 23 | 16.5 |
|  | Prototypic LumB | 12 | 8.6 |
|  | Prototypic Normal-like | 12 | 8.6 |
|  | No | 93 |  |

^a^ For all parameters, numbers are calculated excluding missing data.

**Table S2. Subtype prediction by different strategies on UNC ER+ subset (n=107).***

prototypic ER noTransformation geneCentering subgroupSpecific

H1A7411_0084_C Basal ER+ Normal Basal Basal

H1AUNC_1269_C Basal ER+ Basal Basal Basal

H1AUNC_1304_C Basal ER+ Basal Basal Basal

H1AUNC_1467_C Basal ER+ Normal Basal Basal

H1AUNC_1497C Basal ER+ Normal Basal Basal

H1AUNC_1509_C Basal ER+ Normal Basal Basal

H1A9529_0044C Her2 ER+ LumA Basal Basal

H1AUNC_11483_C Her2 ER+ LumA Her2 LumB

H1AUNC_1211C Her2 ER+ LumA Her2 LumB

H1AUNC_1258 Her2 ER+ LumA Her2 Her2

H1AUNC_1259 Her2 ER+ LumA Her2 Her2

H1AUNC_1265 Her2 ER+ LumA Her2 LumB

H1AUNC_1270_C Her2 ER+ LumA Basal Her2

H1AUNC_1272_C Her2 ER+ LumA LumB LumB

H1AUNC_1276_C Her2 ER+ LumA Her2 Her2

H1AUNC_1280_C Her2 ER+ LumA Her2 Her2

H1AUNC_1318_C Her2 ER+ LumA Her2 Her2

H1AUNC_1465_C Her2 ER+ LumA Her2 Her2

H1AUNC_1488C Her2 ER+ LumA Her2 Her2

H1AUNC_1501C Her2 ER+ LumA Her2 Her2

H1AUNC_1510_C Her2 ER+ LumA Her2 Her2

H1AUNC_1568_C Her2 ER+ LumA Her2 Her2

H1A6261_1499V2 LumA ER+ LumA LumA LumA

H1A6433_2273_V2 LumA ER+ LumA Normal LumA

H1A6433_2978_V2 LumA ER+ LumA Normal LumA

H1A7411_0108_C LumA ER+ LumA Normal LumA

H1A7411_0113_C LumA ER+ LumA LumA LumA

H1A7411_0118_C LumA ER+ LumA LumA LumA

H1A9529_0043C LumA ER+ LumA Normal LumA

H1AUNC_1091_Na LumA ER+ LumA LumA LumA

H1AUNC1166C LumA ER+ LumA LumA LumA

H1AUNC1168C LumA ER+ LumA Basal LumB

H1AUNC1173C LumA ER+ LumA Normal LumA

H1AUNC1176C LumA ER+ LumA LumA LumA

H1AUNC_1200 LumA ER+ LumA Normal LumA

H1AUNC_1305_C LumA ER+ LumA Normal LumA

H1AUNC_1499C LumA ER+ LumA LumA LumA

H1AUNC_1502C LumA ER+ LumA LumA LumA

H1AUNC_1503C LumA ER+ LumA LumA LumA

H1AUNC_1507_C LumA ER+ LumA LumA LumA

H1AUNC_1518_C LumA ER+ LumA LumA LumA

H1AUNC_1566C LumA ER+ LumA Normal LumA

H1A7411_0115_C LumB ER+ LumA Basal LumB

H1AUNC_1088_C_Na LumB ER+ LumA LumB LumB

H1AUNC1171C LumB ER+ LumA LumB LumB

H1AUNC_1268_C LumB ER+ LumA LumB LumB

H1AUNC_1303_C LumB ER+ LumA Her2 LumB

H1AUNC_1319_C LumB ER+ LumB LumB LumB

H1AUNC_1323_C LumB ER+ LumA LumB LumB

H1AUNC_1462_C LumB ER+ LumA LumB LumB

H1AUNC_1471_C LumB ER+ LumA LumB LumB

H1AUNC_1474_C LumB ER+ LumA Basal LumB

H1AUNC_1479_C LumB ER+ LumA LumB LumB

H1AUNC_1571_C LumB ER+ LumA LumB LumB

H1A6261_1500V2 <NA> ER+ LumA LumA LumA

H1A6433_2272V2 <NA> ER+ LumA Her2 LumB

H1A6433_2274_V2 <NA> ER+ LumA LumA LumA

H1A6433_2990V2 <NA> ER+ LumA LumA LumA

H1A6433_3011_V2 <NA> ER+ LumA Normal LumA

H1A6989_8211_V2 <NA> ER+ LumA LumB LumB

H1A6989_8215_V2 <NA> ER+ LumA Normal LumA

H1A6989_8226_V2 <NA> ER+ LumA Normal LumA

H1A7411_0120_C <NA> ER+ LumA LumB LumA

H1A9529_0046C <NA> ER+ LumA Normal LumA

H1A9529_0056C <NA> ER+ LumA Her2 LumB

H1A9529_0063C <NA> ER+ LumA Basal LumA

H1A9529_0065C <NA> ER+ LumA Her2 LumB

H1A9529_025C_Na <NA> ER+ LumA Normal LumA

H1AUNC_0762_C_Na <NA> ER+ LumA LumA LumA

H1AUNC_0763_C_Na <NA> ER+ LumA Normal Normal

H1AUNC_0765_C_Na <NA> ER+ LumA Normal Normal

H1AUNC_1039_C <NA> ER+ LumA LumB LumB

H1AUNC_1117C <NA> ER+ LumA LumA LumA

H1AUNC_1125C <NA> ER+ LumA Her2 LumB

H1AUNC_1197 <NA> ER+ LumA LumA LumA

H1AUNC_1201 <NA> ER+ LumA LumA LumA

H1AUNC_1202 <NA> ER+ LumA LumA LumA

H1AUNC_1260 <NA> ER+ LumA LumB LumB

H1AUNC_1267 <NA> ER+ LumA LumA LumA

H1AUNC_1273_C <NA> ER+ LumA Basal Basal

H1AUNC_1275_C <NA> ER+ LumA LumA LumA

H1AUNC_1278_C <NA> ER+ LumA LumA LumA

H1AUNC_1279_C <NA> ER+ LumA LumA LumA

H1AUNC_1282_C <NA> ER+ LumA LumB LumA

H1AUNC_1283_C <NA> ER+ LumA LumB LumB

H1AUNC_1284_C <NA> ER+ LumA LumA LumA

H1AUNC_1285_C <NA> ER+ LumA LumB LumA

H1AUNC_1301_C <NA> ER+ LumA Basal LumA

H1AUNC_1447_C <NA> ER+ LumA LumB LumA

H1AUNC_1451_C <NA> ER+ LumA LumA LumA

H1AUNC_1459_C <NA> ER+ LumA Her2 LumA

H1AUNC_1466_C <NA> ER+ LumA LumB LumB

H1AUNC_1470_C <NA> ER+ LumA LumB LumB

H1AUNC_1473_C <NA> ER+ LumA Her2 LumA

H1AUNC_1476_C <NA> ER+ LumA LumA LumA

H1AUNC_1490C <NA> ER+ LumA LumB LumB

H1AUNC_1492C <NA> ER+ LumA Normal LumA

H1AUNC_1494C <NA> ER+ LumA Normal LumA

H1AUNC_1495C <NA> ER+ LumA LumA LumA

H1AUNC_1496C <NA> ER+ LumA Her2 LumB

H1AUNC_1504C <NA> ER+ LumA Normal LumA

H1AUNC_1505C <NA> ER+ LumA LumA LumA

H1AUNC_1515_C <NA> ER+ LumA LumA LumA

H1AUNC_1517_C <NA> ER+ LumA LumB LumB

H1AUNC_1520_C <NA> ER+ LumA Normal LumA

H1AUNC_1522_C <NA> ER+ LumA LumB LumA

H1AUNC_1523_C <NA> ER+ LumA LumA LumA

***Footnote: Summary of data in Table S2.**

***Prototypic subtypes (#):***

Basal Her2 LumA LumB

6 16 20 12

***Prototypic subtypes (%):***

Basal Her2 LumA LumB

11 30 37 22

Standard Gene Centering

subtype Basal Her2 LumA LumB Normal

Basal 6 0 0 0 0

Her2 2 13 0 1 0

LumA 1 0 11 0 8

LumB 2 1 0 9 0

Error count: 15

Subgroup Specific Gene Centering

subtype Basal Her2 LumA LumB Normal

Basal 6 0 0 0 0

Her2 1 11 0 4 0

LumA 0 0 19 1 0

LumB 0 0 0 12 0

Error count: 6

***McNemar's Chi-squared test:***

subgroupSpecific_prediction

geneCentering_prediction correct incorrect

correct 36 3

incorrect 12 3

McNemar's chi-squared = 4.2667, df = 1, p-value = 0.03887

**Table S3. Subtype prediction by different strategies on UNC ER- subset (n=71).****

prototypic ER noTransformation geneCentering subgroupSpecific

H1A6261_2597V2_Na Basal ER- Basal Basal Basal

H1A6261_2598V2_Na Basal ER- Normal LumB Basal

H1A6433_2608V2_Na Basal ER- Normal Basal Basal

H1A6433_2610V2_Na Basal ER- Her2 Her2 Basal

H1A6433_3009_V2 Basal ER- Normal Normal Basal

H1A6433_3010_V2 Basal ER- Normal Basal Basal

H1A7411_0111_C Basal ER- Normal Basal Basal

H1A9529_0044Na_C Basal ER- Basal Basal Basal

H1A9529_0061C Basal ER- Normal Basal Basal

H1AUNC_1018_C Basal ER- Normal Normal Basal

H1AUNC_1069_C Basal ER- Normal Basal Basal

H1AUNC_1070_C Basal ER- Normal Basal Basal

H1AUNC_1071_C Basal ER- Normal Normal Basal

H1AUNC_1097_Na Basal ER- Normal LumA Normal

H1AUNC_1122C Basal ER- Normal LumB Basal

H1AUNC__1132_C Basal ER- Normal Basal Basal

H1AUNC_1198 Basal ER- Normal LumB Basal

H1AUNC_1199 Basal ER- Normal Basal Basal

H1AUNC_1212C Basal ER- Normal Basal Basal

H1AUNC_1215C Basal ER- Basal Basal Basal

H1AUNC_1242_C Basal ER- Basal Basal Basal

H1AUNC_1257 Basal ER- Normal Normal Basal

H1AUNC_1271_C Basal ER- Normal Normal Basal

H1AUNC_1274_C Basal ER- Basal Basal Basal

H1AUNC_1277_C Basal ER- Normal Basal Basal

H1AUNC_1281_C Basal ER- Normal Basal Basal

H1AUNC_1321_C Basal ER- Normal Basal Basal

H1AUNC_1446_C Basal ER- Normal Normal Basal

H1AUNC_1448_C Basal ER- Normal Normal Basal

H1AUNC_1449_C Basal ER- Normal Basal Basal

H1AUNC_1450_C Basal ER- Basal Basal Basal

H1AUNC_1456_C Basal ER- Normal Normal Basal

H1AUNC_1458_C Basal ER- Normal Normal Basal

H1AUNC_1463_C Basal ER- Normal Basal Basal

H1AUNC_1468_C Basal ER- Normal Basal Basal

H1AUNC_1469_C Basal ER- Her2 LumB Basal

H1AUNC_1475_C Basal ER- Normal Basal Basal

H1AUNC_1477_C Basal ER- Normal Basal Basal

H1AUNC_1491C Basal ER- Basal Basal Basal

H1AUNC_1493C Basal ER- Normal Normal Basal

H1AUNC_1500C Basal ER- Basal Basal Basal

H1AUNC_1512_C Basal ER- Normal Normal Basal

H1AUNC_1513_C Basal ER- Normal Basal Basal

H1AUNC_1519_C Basal ER- Normal Basal Basal

H1AUNC_1525_C Basal ER- Normal Basal Basal

H1AUNC_1567C Basal ER- Normal Normal Basal

H1A6433_2275_V2 Her2 ER- LumA LumA Her2

H1A6433_2812_V2 Her2 ER- LumA LumA Her2

H1A7411_0114_C Her2 ER- Her2 Her2 Her2

H1A9529_0045C Her2 ER- LumA LumA Her2

H1A9529_0064C Her2 ER- LumA Her2 Her2

H1AUNC_1072_C Her2 ER- LumA LumA Her2

H1AUNC_1118C Her2 ER- Her2 Her2 Her2

H1AUNC_11481_C Her2 ER- LumA LumA LumA

H1AUNC_11484_C Her2 ER- LumA LumA Her2

H1AUNC_1207C Her2 ER- LumA Her2 Her2

H1AUNC_1208C Her2 ER- LumA LumB LumB

H1AUNC_1209C Her2 ER- LumA Her2 Her2

H1AUNC_1266 Her2 ER- LumA LumA Her2

H1AUNC_1316_C Her2 ER- Her2 Her2 Her2

H1AUNC_1324_C Her2 ER- Her2 Her2 Her2

H1AUNC_1489C Her2 ER- LumA Her2 Her2

H1AUNC_1263 LumA ER- LumA LumA LumA

H1AUNC_1487C LumA ER- LumA LumA LumA

H1A9529_0042Na_C <NA> ER- LumA LumA Normal

H1A6433_2979_V2 <NA> ER- Normal LumA Basal

H1AUNC_1124C <NA> ER- LumA LumA LumA

H1AUNC_1235_Na <NA> ER- Her2 LumB LumB

H1AUNC_1261 <NA> ER- LumA LumB Basal

H1AUNC_1302_C <NA> ER- LumA LumA LumA

H1AUNC_1464_C <NA> ER- LumA LumA LumA

**Footnote: Summary of data in Table S3.

***Prototypic subtypes (#):***

Basal Her2 LumA

46 16 2

***Prototypic subtypes (%):***

Basal Her2 LumA

72 25 3

geneCentering

subtype Basal Her2 LumA LumB Normal

Basal 28 1 1 4 12

Her2 0 8 7 1 0

LumA 0 0 2 0 0

Error count: 26

subgroupSpecific

subtype Basal Her2 LumA LumB Normal

Basal 45 0 0 0 1

Her2 0 14 1 1 0

LumA 0 0 2 0 0

Error count: 3

***McNemar's Chi-squared test:***

subgroupSpecific_prediction

geneCentering_prediction correct incorrect

correct 38 0

incorrect 23 3

McNemar's chi-squared = 21.0435, df = 1, p-value = 4.49e-06

**Table S4. Subtype prediction by different strategies on UNC TN subset (n=10).*****

prototypic ER TN noTransformation geneCentering subgroupSpecific

H1A6433_2608V2_Na Basal ER- TN Normal Basal Basal

H1A6433_2610V2_Na Basal ER- TN Her2 Her2 Basal

H1A6433_3010_V2 Basal ER- TN Normal Normal Basal

H1A7411_0111_C Basal ER- TN Normal LumB Basal

H1A9529_0044Na_C Basal ER- TN Basal Basal Basal

H1A9529_0061C Basal ER- TN Normal Basal Basal

H1AUNC_1018_C Basal ER- TN Normal LumA Basal

H1AUNC_1097_Na Basal ER- TN Normal LumA Normal

H1A7411_0114_C Her2 ER- TN Her2 Her2 Her2

H1AUNC_1124C <NA> ER- TN LumA LumA LumA

**Footnote: Summary of data in Table S4.

***Prototypic subtypes (#):***

Basal Her2

8 1

***Prototypic subtypes (%):***

Basal Her2

89 11

geneCentering

subtype Basal Her2 LumA LumB Normal

Basal 3 1 2 1 1

Her2 0 1 0 0 0

Error count: 5

subgroupSpecific

subtype Basal Her2 LumA Normal

Basal 7 0 0 1

Her2 0 1 0 0

Error count: 1

***McNemar's Chi-squared test:***

subgroupSpecific_prediction

geneCentering_prediction correct incorrect

correct 4 0

incorrect 4 1

McNemar's chi-squared = 2.25, df = 1, p-value = 0.1336

**Table S5.** Subtype prediction by different strategies on TCGA triple-negative subgroup (TNBC set; n=77), comparing with the published subtype calls on TCGA cohort.

***Subtyping prediction by gene centering vs published subtype calls:***

geneCentering

publishedSubtype Basal Her2 LumA LumB Normal

Basal 28 5 14 10 8

Her2 0 4 0 1 0

LumA 0 0 3 1 0

LumB 0 0 1 0 0

Normal 0 0 1 0 1

Error count: 41

***Subtyping prediction by subgroup-specific gene centering vs published subtype calls:***

subgroupSpecific

publishedSubtype Basal Her2 LumA LumB Normal

Basal 63 1 0 0 1

Her2 0 5 0 0 0

LumA 0 0 2 2 0

LumB 0 0 0 1 0

Normal 0 0 1 0 1

Error count: 5

***McNemar's Chi-squared test:***

subgroupSpecific_prediction

geneCentering_prediction correct incorrect

correct 35 1

incorrect 37 4

McNemar's chi-squared = 32.2368, df = 1, p-value = 1.365e-08

## Table S6. PAM50 subgroup-specific gene centering baseline value for a few standard subgroups for breast tumors, computed using the UNC training set.

| **Gene** | **Basal-like** | **Her2-enriched** | **LumA** | **LumB** | **Normal-like** | **ER_negative** | **ER_positive** | **TripleNegative** |
| --- | --- | --- | --- | --- | --- | --- | --- | --- |
| ACTR3B | 0.053 | 0.8 | 0.652 | 0.667 | 0.333 | 0.31 | 0.626 | 0.2 |
| ANLN | 0.105 | 0.257 | 0.957 | 0.5 | 1 | 0.183 | 0.667 | 0.3 |
| BAG1 | 0.754 | 0.8 | 0.13 | 0.667 | 0.167 | 0.718 | 0.402 | 0.8 |
| BCL2 | 0.474 | 0.571 | 0.522 | 0.917 | 0.333 | 0.493 | 0.579 | 0.5 |
| BIRC5 | 0.157 | 0.242 | 0.95 | 0.083 | 0.917 | 0.258 | 0.646 | 0.143 |
| BLVRA | 0.842 | 0.171 | 0.435 | 0 | 0.833 | 0.704 | 0.271 | 0.8 |
| CCNB1 | 0.175 | 0.314 | 0.913 | 0 | 1 | 0.254 | 0.598 | 0.3 |
| CCNE1 | 0.035 | 0.343 | 0.913 | 0.5 | 0.917 | 0.127 | 0.692 | 0.1 |
| CDC20 | 0.07 | 0.429 | 0.957 | 0.5 | 0.583 | 0.197 | 0.71 | 0.2 |
| CDC6 | 0.175 | 0.2 | 0.95 | 0 | 1 | 0.243 | 0.594 | 0.2 |
| CDCA1 | 0.088 | 0.429 | 0.864 | 0.083 | 0.833 | 0.211 | 0.621 | 0.1 |
| CDH3 | 0.14 | 0.343 | 0.826 | 1 | 0 | 0.257 | 0.748 | 0.4 |
| CENPF | 0.035 | 0.371 | 0.783 | 0.25 | 0.75 | 0.169 | 0.645 | 0.1 |
| CEP55 | 0.053 | 0.314 | 0.957 | 0.167 | 1 | 0.141 | 0.667 | 0.1 |
| CXXC5 | 0.965 | 0.543 | 0.304 | 0.167 | 0.417 | 0.831 | 0.299 | 0.9 |
| EGFR | 0.263 | 0.543 | 0.696 | 0.75 | 0.167 | 0.286 | 0.664 | 0.4 |
| ERBB2 | 0.807 | 0.286 | 0.261 | 0.417 | 1 | 0.676 | 0.393 | 0.9 |
| ESR1 | 1 | 0.735 | 0 | 0.083 | 0.833 | 0.906 | 0.2 | 0.9 |
| EXO1 | 0.088 | 0.343 | 0.81 | 0.333 | 0.909 | 0.229 | 0.643 | 0.2 |
| FGFR4 | 0.725 | 0.182 | 0.6 | 0.583 | 0 | 0.541 | 0.604 | 0.286 |
| FOXA1 | 1 | 0.486 | 0.043 | 0.25 | 1 | 0.765 | 0.231 | 0.889 |
| FOXC1 | 0.035 | 0.886 | 0.609 | 0.917 | 0 | 0.282 | 0.766 | 0.3 |
| GPR160 | 0.929 | 0.286 | 0.261 | 0.167 | 0.917 | 0.725 | 0.28 | 0.889 |
| GRB7 | 0.596 | 0.229 | 0.609 | 0.5 | 0.25 | 0.549 | 0.495 | 0.8 |
| KIF2C | 0.14 | 0.514 | 0.739 | 0.5 | 0.75 | 0.239 | 0.664 | 0.2 |
| KNTC2 | 0.035 | 0.343 | 0.957 | 0.25 | 1 | 0.183 | 0.664 | 0.2 |
| KRT14 | 0.368 | 0.8 | 0.304 | 1 | 0 | 0.465 | 0.645 | 0.6 |
| KRT17 | 0.351 | 0.571 | 0.478 | 1 | 0 | 0.451 | 0.673 | 0.4 |
| KRT5 | 0.316 | 0.771 | 0.522 | 0.917 | 0 | 0.423 | 0.664 | 0.4 |
| MAPT | 0.754 | 0.714 | 0.13 | 0.583 | 0.417 | 0.786 | 0.352 | 0.8 |
| MDM2 | 0.509 | 0.6 | 0.522 | 0.727 | 0.333 | 0.486 | 0.505 | 0.4 |
| MELK | 0.123 | 0.286 | 0.909 | 0.167 | 1 | 0.197 | 0.644 | 0.2 |
| MIA | 0.123 | 0.781 | 0.739 | 1 | 0 | 0.333 | 0.763 | 0.4 |
| MKI67 | 0.105 | 0.457 | 0.818 | 0.5 | 0.75 | 0.225 | 0.638 | 0.1 |
| MLPH | 0.725 | 0.667 | 0.2 | 0.833 | 0.083 | 0.694 | 0.469 | 0.714 |
| MMP11 | 0.614 | 0.314 | 0.565 | 0.25 | 1 | 0.543 | 0.368 | 0.6 |
| MYBL2 | 0.035 | 0.286 | 0.957 | 0.25 | 1 | 0.183 | 0.692 | 0.2 |
| MYC | 0.211 | 0.829 | 0.652 | 0.417 | 0.083 | 0.366 | 0.664 | 0.3 |
| NAT1 | 0.964 | 0.571 | 0.043 | 0.417 | 0.75 | 0.838 | 0.234 | 0.9 |
| ORC6L | 0.148 | 0.367 | 0.765 | 0.636 | 0.9 | 0.242 | 0.663 | 0.111 |
| PGR | 0.647 | 0.515 | 0.35 | 0.75 | 0.5 | 0.607 | 0.448 | 0.857 |
| PHGDH | 0.07 | 0.457 | 0.783 | 0.917 | 0.083 | 0.211 | 0.766 | 0.1 |
| PTTG1 | 0.07 | 0.257 | 1 | 0.25 | 0.917 | 0.155 | 0.654 | 0.2 |
| RRM2 | 0.14 | 0.114 | 0.957 | 0.083 | 1 | 0.197 | 0.645 | 0.3 |
| SFRP1 | 0.14 | 0.857 | 0.478 | 0.917 | 0 | 0.31 | 0.755 | 0.2 |
| SLC39A6 | 0.93 | 0.8 | 0 | 0.167 | 0.417 | 0.873 | 0.243 | 1 |
| TMEM45B | 0.961 | 0.114 | 0.304 | 0.2 | 0.583 | 0.688 | 0.356 | 0.7 |
| TYMS | 0.105 | 0.4 | 0.957 | 0.083 | 1 | 0.225 | 0.626 | 0.3 |
| UBE2C | 0.089 | 0.371 | 0.957 | 0.083 | 0.917 | 0.229 | 0.598 | 0.2 |
| UBE2T | 0.07 | 0.257 | 0.957 | 0 | 1 | 0.183 | 0.589 | 0.3 |
