# Supplementary figures and images for: Molecular subtyping for clinically defined breast cancer subgroups
Source: Breast Cancer Res. 2015 Feb 26;17(1):29. doi: 10.1186/s13058-015-0520-4 (PMC4365540; doi:10.1186/s13058-015-0520-4)

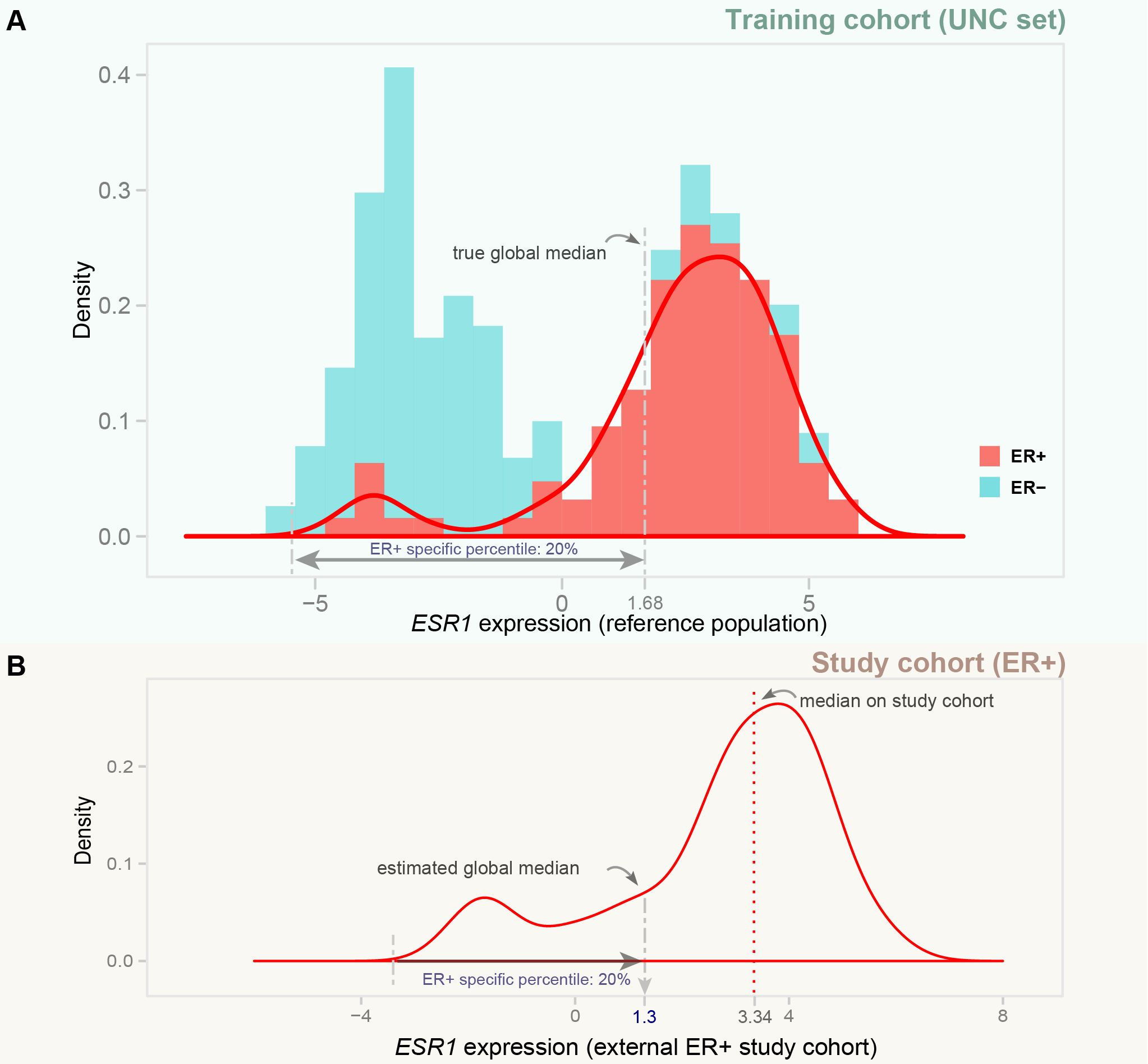

Supplement: Additional file 2: Figure S1. — Subgroup-specific strategy for molecular subtype assignment in an ER-positive study cohort (Trondheim set) demonstrated for a single gene, ESR1. (A) Computing probe-wise subgroup percentiles. On the training cohort (UNC set), the global median (indicated by the gray dashed line) is mapped onto the distribution of ER-positive subgroup, and the corresponding percentile (20%) is the ER-positive-specific percentile used to adjust the expression of the gene ESR1 in the study cohort. (B) Probe-wise transformation using subgroup-specific gene centering. On an external ER-positive study cohort (Trondheim set), instead of centering the gene value on the median of the study cohort, which is 3.34, as indicated by the red vertical dotted line), we center the gene ESR1 around the 20th percentile of its distribution in the study cohort, which is 1.3. [file 13058_2015_520_MOESM2_ESM.tiff]

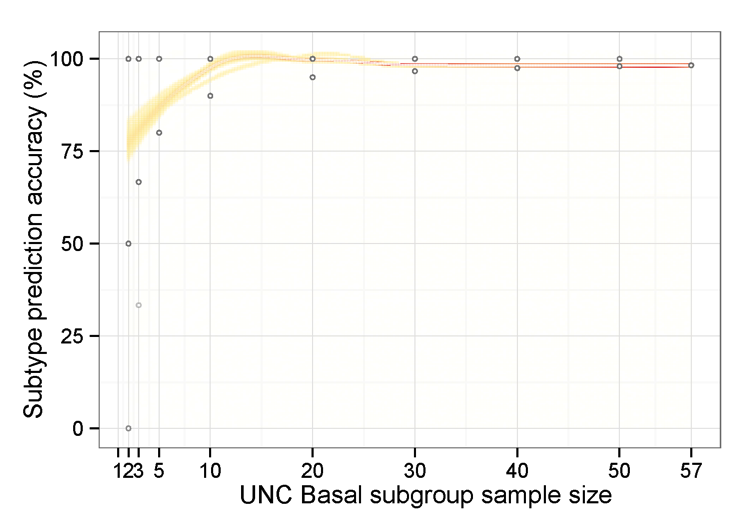

Supplement: Additional file 4: Figure S3. — Subtyping accuracy versus subgroup sample size of UNC basal prototypical subgroup. The horizontal axis denotes the sample size of data from downsampling UNC basal prototypical subgroup (from 57 to 1). The vertical axis shows the subtyping prediction accuracy using our subgroup-specific gene centering method. The accuracy is calculated based on the percentage of the predicted basal subtype on the corresponding dataset. Individual predictions are shown as circles, and a Loess smooth line (span 0.75) is fitted on the prediction points. Two hundred rounds of downsampling were performed. The color of the lines reflects the statistical confidence in the regression estimation. [file 13058_2015_520_MOESM4_ESM.png]

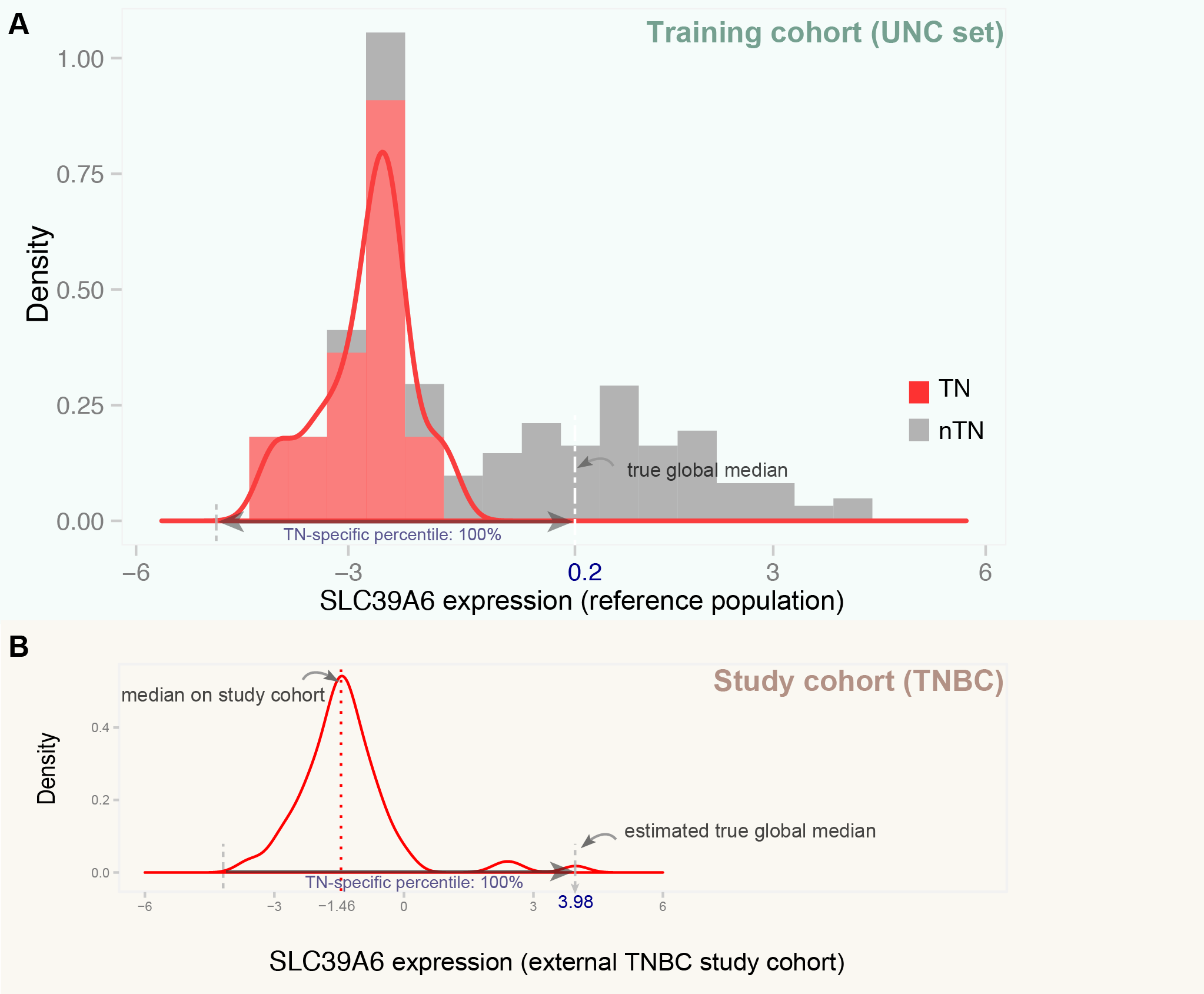

Supplement: Additional file 5: Figure S2. — Subgroup-specific strategy for molecular subtype assignment in a triple-negative breast cancer study cohort for the expression of gene SLC39A6. (A) Computing probe-wise subgroup percentiles. On the training cohort (UNC set), the global median of the UNC set (indicated by the white dashed line) is mapped onto the distribution of the TN subgroup, and the corresponding percentile (100%) is the TN-specific percentile for gene SLC39A6. (B) Probe-wise transformation using subgroup-specific percentile. On an external TNBC study cohort (TNBC set), instead of centering on the median of the study cohort (−1.46), we centered the distribution of gene SLC39A6 on the 100% percentile of the TN distribution of the study cohort (3.98). [file 13058_2015_520_MOESM5_ESM.tiff]
