# Supplementary material for: Molecular subtyping for clinically defined breast cancer subgroups
Source: Breast Cancer Res. 2015 Feb 26;17(1):29. doi: 10.1186/s13058-015-0520-4 (PMC4365540; doi:10.1186/s13058-015-0520-4)
Supplement: Additional file 3: — Supplementary methods. Molecular subtyping for clinically defined breast cancer subgroups. [file 13058_2015_520_MOESM3_ESM.doc]

Supplementary Methods

**Molecular subtyping for clinically-defined breast cancer subgroups**

Xi Zhao, Einar Andreas Rødland, Robert Tibshirani, Sylvia Plevritis.

Table of Contents

Supplementary Methods [2](#__RefHeading___Toc275726923)

1. Data [2](#__RefHeading___Toc275726924)

*1.1* *UNC dataset* [2](#__RefHeading___Toc275726925)

*1.2* *TNBC set* [2](#__RefHeading___Toc275726926)

*1.3* *Trondheim set* [2](#__RefHeading___Toc275726927)

2. Gene annotation mapping [2](#__RefHeading___Toc275726928)

3. Statistical analysis [3](#__RefHeading___Toc275726929)

*3.1* *Gene expression molecular subtyping* [3](#__RefHeading___Toc275726930)

*3.2* *Standard gene centering* [3](#__RefHeading___Toc275726931)

*3.3* *Subgroup-specific gene centering* [3](#__RefHeading___Toc275726932)

*3.4* *Software* [5](#__RefHeading___Toc275726933)

4. Sensitivity Analysis [5](#__RefHeading___Toc275726934)

5. References [6](#__RefHeading___Toc275726935)

# Supplementary Methods

## Data

### *UNC dataset*

The UNC gene expression data was downloaded from <https://genome.unc.edu/pubsup/breastGEO/pam50_arrayPrototypes.xls> (Accessed on July 16, 2012). The clinical metadata for the UNC training dataset was retrieved from Gene Expression Omnibus (http://www.ncbi.nlm.nih.gov/geo) with accession number GSE10886 and <https://genome.unc.edu/pubsup/breastGEO/clinicalData.shtml> (Accessed on July 16, 2012).

### *TNBC set*

The TCGA breast level 3 data, its clinical annotation and the published molecular subtype calls were obtained through the TCGA website (http://cancergenome. nih.gov).

### *Trondheim set*

Expression profiling of a set of 48 ER-positive breast tumors was performed on 44k two-color Agilent Human Whole Genome Oligo Microarrays. Procedure for data preprocessing and normalization is described in the original study .

## Gene annotation mapping

The TNBC expression set was annotated using gene symbols, which were used to match to PAM50 signature. The original Agilent probes in the Trondheim data were matched against gene symbols of the PAM50 using BioMart through R library biomaRt (Ensembl release 67/ hg19 human assembly). For the probes with the same gene symbols, we selected probe(s) with the largest interquartile range (IQR: difference between the third and first quartiles) among the multiple hits. If there were still more than one hit per gene symbol, we further averaged the expression values of those probes for each sample.

We achieved 94% (47/50) cross-platform gene annotation mapping coverage for PAM50 on TNBC set and 92% (46/50) coverage on the Trondheim ER-positive dataset.

## Statistical analysis

### *Gene expression molecular subtyping*

In this study, the PAM50 molecular subtype classifier was used for subtype classification. The molecular subtype is assigned to each individual tumor within a study cohort to its closest matched subtype expression profile using the nearest centroid approach, where distances are measured by the Spearman correlations to the subtype centroids.

### *Standard gene centering*

The median of a specific gene across all samples of the study cohort was subtracted from that gene, followed by the nearest centroid approach on the transformed data for subtype assignments.

### *Subgroup-specific gene centering*

To perform subtype classification on a skewed dataset, e.g. a clinical subgroup of cancer, the key is to accurately estimate the average expression of individual genes in a population similar to the training cohort which was used to develop the signatures. This represents the expression baseline of each gene or probe, and can be estimated as the average expression in a heterogeneous population. However, for a skewed data or tumor subgroup, this gene expression baseline will generally not be the sample average, but can instead be estimated in terms of the appropriate percentile within the sample (Figure 1, main text). Thus, instead of gene centering by subtracting the average gene expression from within the sample, for each gene the expression is centered against this percentile. By utilizing these subgroup-dependent percentiles estimated from the training cohort, we are able to infer the location of the expression baselines for individual genes on a new study dataset collected from the corresponding breast caner subgroup of interest.

We proposed a probe-wise subgroup-specific transformation prior to the nearest centroid approach for subtype classification in a skewed study cohort.

The heterogeneous breast cancer population consists of a mix of subgroups. We refer to the population on which a particular subtype signature has been developed as the reference population: for PAM50, this would be the population represented by the UNC set (n = 232). Typically, this reference population would be a heterogeneous sample representative of breast cancer tumors. Given a cohort of tumors which has been sampled from a subgroup of the reference population, e.g. ER-positive tumors, proper gene centering is done in three steps. 1) For each gene in the subtype signature, the reference population average gene expression is estimated, which is be the baseline for gene centering: this may be done using the training cohort on which the subtype signatures were developed, or a different cohort representative of the sample population. 2) On this sample, the subgroup corresponding to the inclusion criteria of the new study cohort is identified, and for each gene we determine which percentile within the subgroup corresponds to the baseline value (See Supplement Table S6 for baseline values we precomputed for common subgroups of breast tumors). 3) In the new study cohort, we find the same percentile, and use that as baseline for gene centering.

Using PAM50 as an example, and the UNC (n = 232) training cohort to represent the reference population, let be the expression vector for *p*th gene of the PAM50 classifier (*p* =1, …, 50) across all *n* patients in the UNC dataset. PAM50 uses the median for gene centering, and so the average expression of the *p*th gene is . Other subtyping methods may specify the mean as the expression average to use for gene centering, in which case the mean should be used instead of the median.

We identify the subgroup of tumors in the training cohort that match the inclusion criteria used in the new study cohort, e.g. ER-positive tumors, and quantify *µ_p_* relative to the distribution of gene expression values in this tumor subgroup. Specifically, the *subgroup-specific percentile* of signature gene *p* in the clinically relevant subgroup, denoted by *Q_p,subgroup_*, is defined as

where F_p_*_, subgroup_* is the cumulative distribution function estimated from the *p*th gene´s expression profiles in this tumor subgroup of the UNC set: . Note that so far we have not used data from the study cohort, only the specified tumor subgroup of interest defined by the inclusion criterion of the study cohort, hence the percentile *Q_p,subgroup_* can be precomputed for different subgroups (Table S6).

In a study cohort with a total of *m* samples representing a given tumor subgroup, e.g. ER-positive, and given the percentiles *Q_p,subgroup_* previously determined for this subgroup, we estimate the gene expression baseline  against which to center gene *p* as the *Q_p,subgroup_* percentile. i.e., if the cumulative distribution function of the *p*th gene is constructed from *.* The gene expression baseline (unobserved global population median) is then estimated as:

Expression values for gene *p* in the new study cohort are then transformed by subtracting :

The above procedure is carried out for all genes of the PAM50 signature. These transformed values are then used to compute centroid correlations, which for PAM50 is done using Spearman correlations, and the subtype assigned corresponding to the centroid with the highest correlation. No threshold was set on the correlation when performing subtyping, that is, every tumor received a subtype call.

***Implementation of subtyping on a mixture study cohort***

If the study cohort itself is a mixture of several defined cohorts (e.g. with 15% ER-positive and 85% ER-negative samples), we perform the normalization based on a subgroup of the training data with a similar mixture as following:

- Step (1): Sample the training cohort with the similar composition of patients characteristics as those in the study cohort (e.g. 15% ER-positive and 85% ER-negative), and compute the subgroup-specific percentile per gene;
- Step (2): Repeat Step 1 B times (e.g. B=50);
- Step (3): Average the computed percentile to generate the subgroup-specific percentile per gene;
- Step (4): Center around the percentile for each gene and perform subtype classification

The sampling procedure is relatively computationally demanding, and there is randomness for extracting the corresponding UNC subgroups at specific proportions. Instead, we can subtype each of the subsets of the study cohort separately using the proposed subgroup-specific method. This nonstochastic approach provides a far more practical alternative for subtyping a mixed cohort.

### *Software*

Our proposed subgroup-specific gene centering method is implemented in R (version 3.0.0) . Data and code were deposited at <http://ccsb.stanford.edu/research/core.html>.

## Sensitivity Analysis

We performed the following a sensitivity analysis to demonstrate the dependence on size of sampled subgroup in the training cohort.

We use Basal prototypical subgroup on UNC dataset to illustrate the relationship between subtyping accuracy and a subgroup sample size (Figure 1; also included as Figure S3). Basal prototypical subgroup was selected because it is the largest prototypical subgroup (n = 57) on UNC set. We down-sampled this subgroup into datasets of size 57, 50, 40, 30, 20, 10, 5, 3, 2, 1, respectively. And the subtyping accuracy was calculated using the percentage of predicted “Basal” label on the tested dataset. This process was then repeated 200 times (200 predictions for a particular tested sample size). Figure 1 shows that we can achieve 80% accuracy with 5 samples in the subgroup of interest, and with 10 samples and beyond, the prediction is stable with almost 100% accuracy. With sample size 3, we observed 128 out of 200 predictions (64%) with 100% accuracy, 67 predictions (33.5%) with 66.7% accuracy and 5 predictions (2.5%) with 33.3% accuracy. Thus, our subtyping method is very likely to give a correct prediction even under sample size 3. When sample size is 1 and centering is carried out (either median centering by conventional method or our subgroup-specific centering will result expression 0 for all PAM50 genes of the sample), it is not possible to obtain a prediction by using correlation. The subtyping could potentially be carried out without any centering. However, given limitation by the platform (as discussed in the main text, see also Figure 3 in the main text), we do not recommend subtyping without any data transformation.

## References

1. Edgar R, Domrachev M, Lash AE: **Gene Expression Omnibus: NCBI gene expression and hybridization array data repository**. *Nucleic acids research* 2002, **30**(1):207.

2. Borgan E, Sitter B, Lingjærde O, Johnsen H, Lundgren S, Bathen T, Sørlie T, Børresen-Dale A-L, Gribbestad I: **Merging transcriptomics and metabolomics-advances in breast cancer profiling**. *BMC Cancer* 2010, **10**(1):628.

3. Parker JS, Mullins M, Cheang MCU, Leung S, Voduc D, Vickery T, Davies S, Fauron C, He X, Hu Z: **Supervised risk predictor of breast cancer based on intrinsic subtypes**. *Journal of Clinical Oncology* 2009, **27**(8):1160.

4. R Development Core Team: **R: A language and environment for statistical computing**. *R Foundation for Statistical Computing* 2011.
